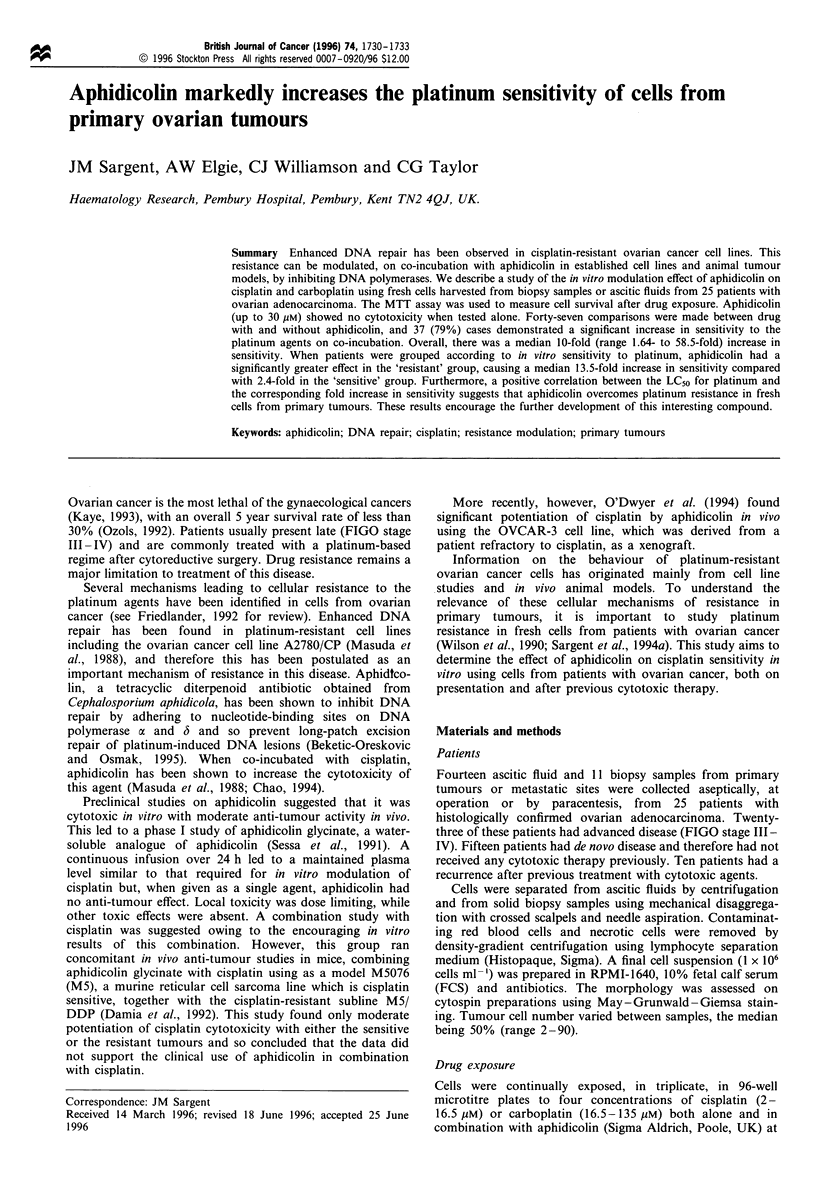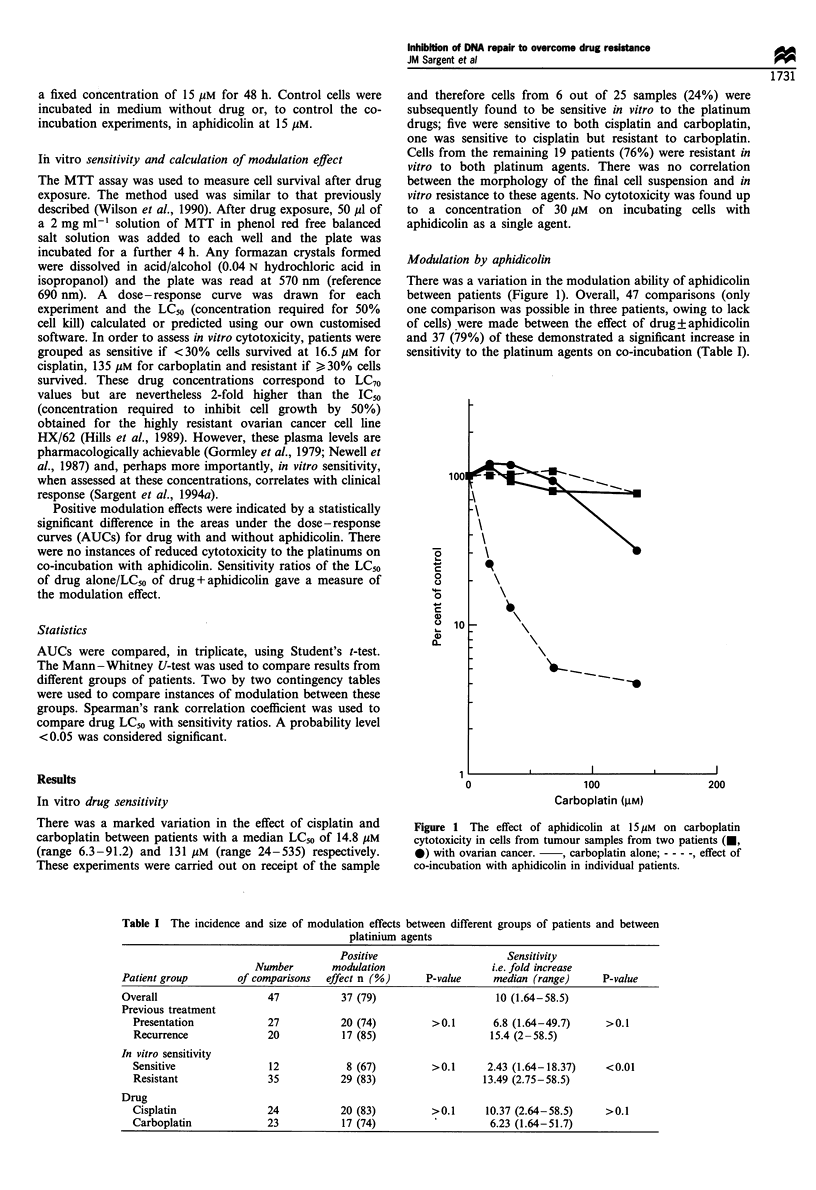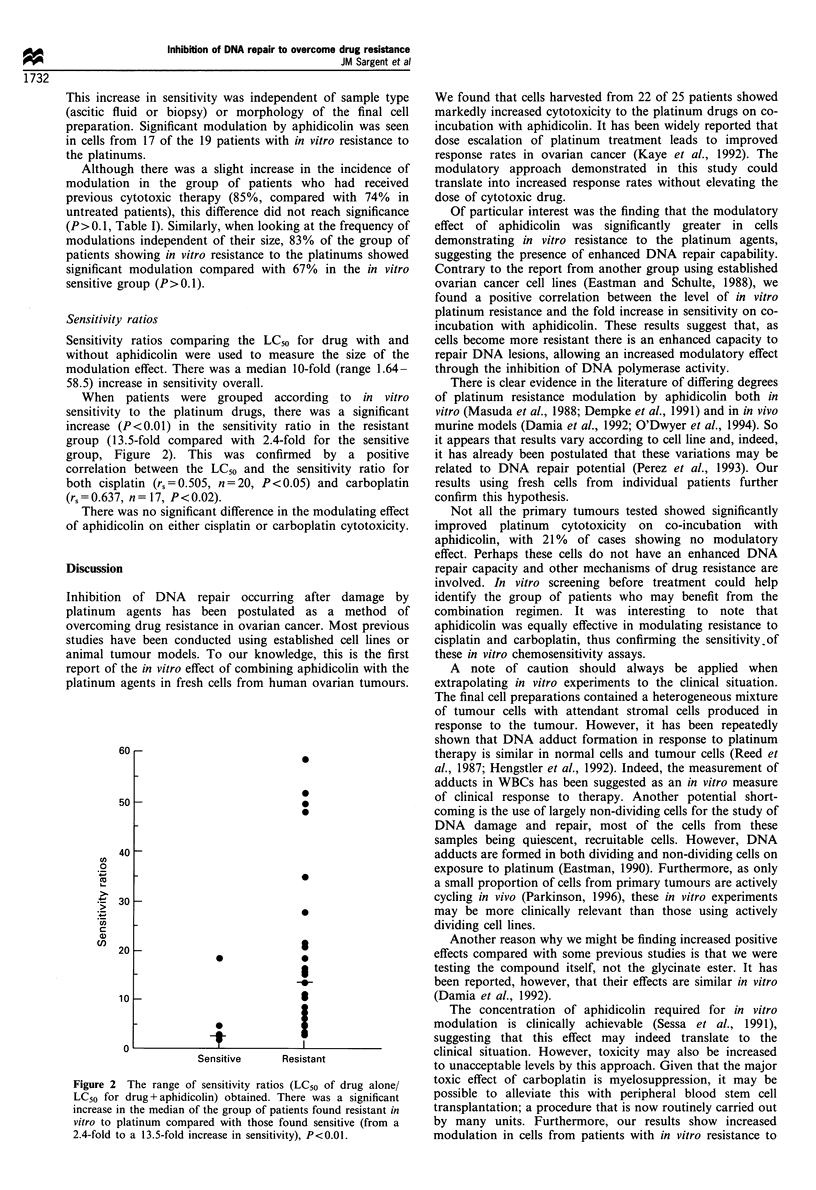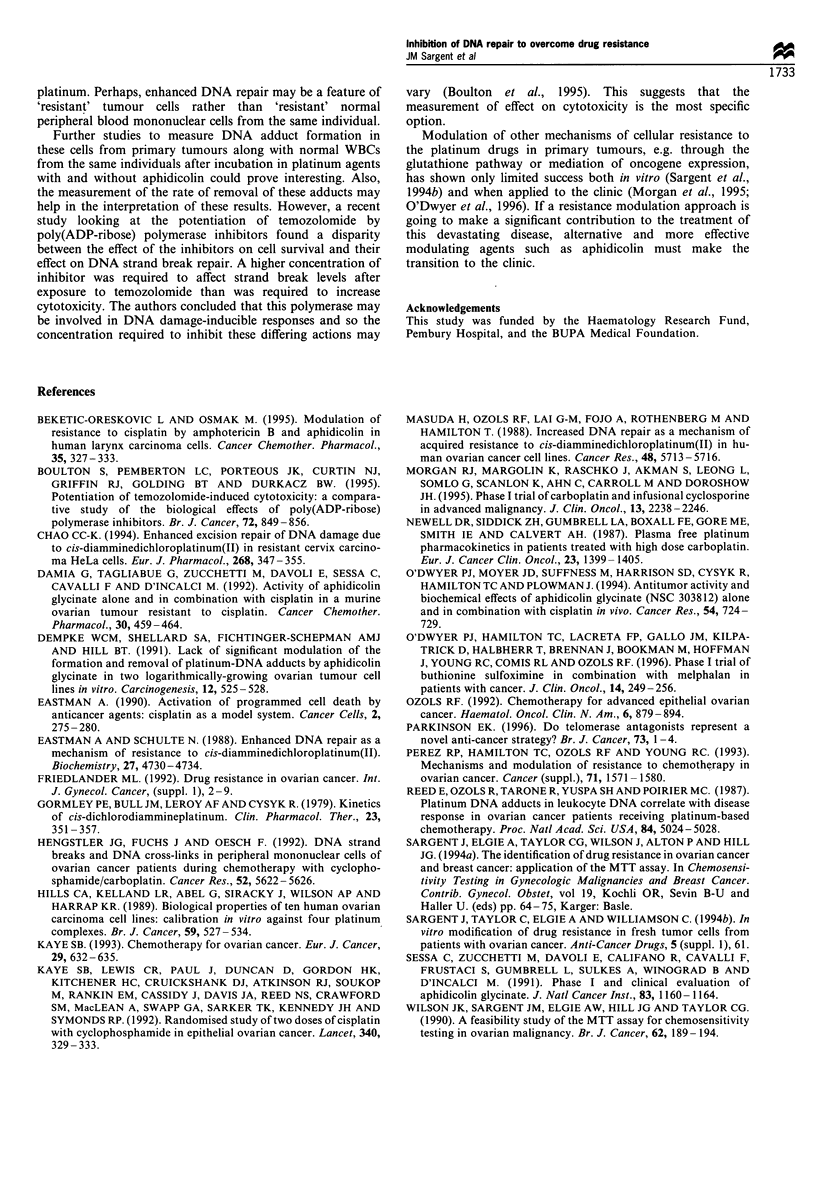# Aphidicolin markedly increases the platinum sensitivity of cells from primary ovarian tumours.

**DOI:** 10.1038/bjc.1996.622

**Published:** 1996-12

**Authors:** J. M. Sargent, A. W. Elgie, C. J. Williamson, C. G. Taylor

**Affiliations:** Pembury Hospital, Kent, UK.

## Abstract

Enhanced DNA repair has been observed in cisplatin-resistant ovarian cancer cell lines. This resistance can be modulated, on co-incubation with aphidicolin in established cell lines and animal tumour models, by inhibiting DNA polymerases. We describe a study of the in vitro modulation effect of aphidicolin on cisplatin and carboplatin using fresh cells harvested from biopsy samples or ascitic fluids from 25 patients with ovarian adenocarcinoma. The MTT assay was used to measure cell survival after drug exposure. Aphidicolin (up to 30 microM) showed no cytotoxicity when tested alone. Forty-seven comparisons were made between drug with and without aphidicolin, and 37 (79%) cases demonstrated a significant increase in sensitivity to the platinum agents on co-incubation. Overall, there was a median 10-fold (range 1.64- to 58.5-fold) increase in sensitivity. When patients were grouped according to in vitro sensitivity to platinum, aphidicolin had a significantly greater effect in the "resistant' group, causing a median 13.5-fold increase in sensitivity compared with 2.4-fold in the "sensitive' group. Furthermore, a positive correlation between the LC50 for platinum and the corresponding fold increase in sensitivity suggests that aphidicolin overcomes platinum resistance in fresh cells from primary tumours. These results encourage the further development of this interesting compound.


British Journal of Cancer (1996) 74, 1730-1733
© 1996 Stockton Press All rights reserved 0007-0920/96 $12.00

### Aphidicolin markedly increases the platinum sensitivity of cells from primary ovarian tumours

JM Sargent, AW Elgie, CJ Williamson and CG Taylor

*Haematology Research, Pembury Hospital, Pembury, Kent TN2 4QJ, UK.*

**Summary** Enhanced DNA repair has been observed in cisplatin-resistant ovarian cancer cell lines. This resistance can be modulated, on co-incubation with aphidicolin in established cell lines and animal tumour models, by inhibiting DNA polymerases. We describe a study of the *in vitro* modulation effect of aphidicolin on cisplatin and carboplatin using fresh cells harvested from biopsy samples or ascitic fluids from 25 patients with ovarian adenocarcinoma. The MTT assay was used to measure cell survival after drug exposure. Aphidicolin (up to 30 $\mu$M) showed no cytotoxicity when tested alone. Forty-seven comparisons were made between drug with and without aphidicolin, and 37 (79%) cases demonstrated a significant increase in sensitivity to the platinum agents on co-incubation. Overall, there was a median 10-fold (range 1.64- to 58.5-fold) increase in sensitivity. When patients were grouped according to *in vitro* sensitivity to platinum, aphidicolin had a significantly greater effect in the 'resistant' group, causing a median 13.5-fold increase in sensitivity compared with 2.4-fold in the 'sensitive' group. Furthermore, a positive correlation between the $LC_{50}$ for platinum and the corresponding fold increase in sensitivity suggests that aphidicolin overcomes platinum resistance in fresh cells from primary tumours. These results encourage the further development of this interesting compound.

**Keywords:** aphidicolin; DNA repair; cisplatin; resistance modulation; primary tumours

Ovarian cancer is the most lethal of the gynaecological cancers (Kaye, 1993), with an overall 5 year survival rate of less than 30% (Ozols, 1992). Patients usually present late (FIGO stage III-IV) and are commonly treated with a platinum-based regime after cytoreductive surgery. Drug resistance remains a major limitation to treatment of this disease.

Several mechanisms leading to cellular resistance to the platinum agents have been identified in cells from ovarian cancer (see Friedlander, 1992 for review). Enhanced DNA repair has been found in platinum-resistant cell lines including the ovarian cancer cell line A2780/CP (Masuda *et al.*, 1988), and therefore this has been postulated as an important mechanism of resistance in this disease. Aphidicolin, a tetracyclic diterpenoid antibiotic obtained from *Cephalosporium aphidicola*, has been shown to inhibit DNA repair by adhering to nucleotide-binding sites on DNA polymerase $\alpha$ and $\delta$ and so prevent long-patch excision repair of platinum-induced DNA lesions (Beketic-Oreskovic and Osmak, 1995). When co-incubated with cisplatin, aphidicolin has been shown to increase the cytotoxicity of this agent (Masuda *et al.*, 1988; Chao, 1994).

Preclinical studies on aphidicolin suggested that it was cytotoxic *in vitro* with moderate anti-tumour activity *in vivo*. This led to a phase I study of aphidicolin glycinate, a water-soluble analogue of aphidicolin (Sessa *et al.*, 1991). A continuous infusion over 24 h led to a maintained plasma level similar to that required for *in vitro* modulation of cisplatin but, when given as a single agent, aphidicolin had no anti-tumour effect. Local toxicity was dose limiting, while other toxic effects were absent. A combination study with cisplatin was suggested owing to the encouraging *in vitro* results of this combination. However, this group ran concomitant *in vivo* anti-tumour studies in mice, combining aphidicolin glycinate with cisplatin using as a model M5076 (M5), a murine reticular cell sarcoma line which is cisplatin sensitive, together with the cisplatin-resistant subline M5/DDP (Damia *et al.*, 1992). This study found only moderate potentiation of cisplatin cytotoxicity with either the sensitive or the resistant tumours and so concluded that the data did not support the clinical use of aphidicolin in combination with cisplatin.

More recently, however, O'Dwyer *et al.* (1994) found significant potentiation of cisplatin by aphidicolin *in vivo* using the OVCAR-3 cell line, which was derived from a patient refractory to cisplatin, as a xenograft.

Information on the behaviour of platinum-resistant ovarian cancer cells has originated mainly from cell line studies and *in vivo* animal models. To understand the relevance of these cellular mechanisms of resistance in primary tumours, it is important to study platinum resistance in fresh cells from patients with ovarian cancer (Wilson *et al.*, 1990; Sargent *et al.*, 1994*a*). This study aims to determine the effect of aphidicolin on cisplatin sensitivity *in vitro* using cells from patients with ovarian cancer, both on presentation and after previous cytotoxic therapy.

### Materials and methods

### *Patients*

Fourteen ascitic fluid and 11 biopsy samples from primary tumours or metastatic sites were collected aseptically, at operation or by paracentesis, from 25 patients with histologically confirmed ovarian adenocarcinoma. Twenty-three of these patients had advanced disease (FIGO stage III-IV). Fifteen patients had *de novo* disease and therefore had not received any cytotoxic therapy previously. Ten patients had a recurrence after previous treatment with cytotoxic agents.

Cells were separated from ascitic fluids by centrifugation and from solid biopsy samples using mechanical disaggregation with crossed scalpels and needle aspiration. Contaminating red blood cells and necrotic cells were removed by density-gradient centrifugation using lymphocyte separation medium (Histopaque, Sigma). A final cell suspension ($1 \times 10^6$ cells $ml^{-1}$) was prepared in RPMI-1640, 10% fetal calf serum (FCS) and antibiotics. The morphology was assessed on cytospin preparations using May-Grunwald-Giemsa staining. Tumour cell number varied between samples, the median being 50% (range 2-90).

### *Drug exposure*

Cells were continually exposed, in triplicate, in 96-well microtitre plates to four concentrations of cisplatin (2-16.5 $\mu$M) or carboplatin (16.5-135 $\mu$M) both alone and in combination with aphidicolin (Sigma Aldrich, Poole, UK) at

Correspondence: JM Sargent
Received 14 March 1996; revised 18 June 1996; accepted 25 June 1996

a fixed concentration of 15 μM for 48 h. Control cells were incubated in medium without drug or, to control the co-incubation experiments, in aphidicolin at 15 μM.

### In vitro *sensitivity and calculation of modulation effect*

The MTT assay was used to measure cell survival after drug exposure. The method used was similar to that previously described (Wilson *et al.*, 1990). After drug exposure, 50 μl of a 2 mg $ml^{-1}$ solution of MTT in phenol red free balanced salt solution was added to each well and the plate was incubated for a further 4 h. Any formazan crystals formed were dissolved in acid/alcohol (0.04 N hydrochloric acid in isopropanol) and the plate was read at 570 nm (reference 690 nm). A dose–response curve was drawn for each experiment and the $LC_{50}$ (concentration required for 50% cell kill) calculated or predicted using our own customised software. In order to assess *in vitro* cytotoxicity, patients were grouped as sensitive if <30% cells survived at 16.5 μM for cisplatin, 135 μM for carboplatin and resistant if ⩾30% cells survived. These drug concentrations correspond to $LC_{70}$ values but are nevertheless 2-fold higher than the $IC_{50}$ (concentration required to inhibit cell growth by 50%) obtained for the highly resistant ovarian cancer cell line HX/62 (Hills *et al.*, 1989). However, these plasma levels are pharmacologically achievable (Gormley *et al.*, 1979; Newell *et al.*, 1987) and, perhaps more importantly, *in vitro* sensitivity, when assessed at these concentrations, correlates with clinical response (Sargent *et al.*, 1994*a*).

Positive modulation effects were indicated by a statistically significant difference in the areas under the dose–response curves (AUCs) for drug with and without aphidicolin. There were no instances of reduced cytotoxicity to the platinums on co-incubation with aphidicolin. Sensitivity ratios of the $LC_{50}$ of drug alone/$LC_{50}$ of drug + aphidicolin gave a measure of the modulation effect.

### *Statistics*

AUCs were compared, in triplicate, using Student's *t*-test. The Mann–Whitney *U*-test was used to compare results from different groups of patients. Two by two contingency tables were used to compare instances of modulation between these groups. Spearman's rank correlation coefficient was used to compare drug $LC_{50}$ with sensitivity ratios. A probability level <0.05 was considered significant.

### Results

### In vitro *drug sensitivity*

There was a marked variation in the effect of cisplatin and carboplatin between patients with a median $LC_{50}$ of 14.8 μM (range 6.3–91.2) and 131 μM (range 24–535) respectively. These experiments were carried out on receipt of the sample and therefore cells from 6 out of 25 samples (24%) were subsequently found to be sensitive *in vitro* to the platinum drugs; five were sensitive to both cisplatin and carboplatin, one was sensitive to cisplatin but resistant to carboplatin. Cells from the remaining 19 patients (76%) were resistant *in vitro* to both platinum agents. There was no correlation between the morphology of the final cell suspension and *in vitro* resistance to these agents. No cytotoxicity was found up to a concentration of 30 μM on incubating cells with aphidicolin as a single agent.

### *Modulation by aphidicolin*

There was a variation in the modulation ability of aphidicolin between patients (Figure 1). Overall, 47 comparisons (only one comparison was possible in three patients, owing to lack of cells) were made between the effect of drug ± aphidicolin and 37 (79%) of these demonstrated a significant increase in sensitivity to the platinum agents on co-incubation (Table I).

**Figure 1** The effect of aphidicolin at 15 μM on carboplatin cytotoxicity in cells from tumour samples from two patients (■, ●) with ovarian cancer. ——, carboplatin alone; - - - -, effect of co-incubation with aphidicolin in individual patients.

**Table I** The incidence and size of modulation effects between different groups of patients and between platinium agents

| *Patient group* | *Number of comparisons* | *Positive modulation effect* n *(%)* | P-*value* | *Sensitivity i.e. fold increase median (range)* | P-*value* |
|---|---|---|---|---|---|
| Overall | 47 | 37 (79) | | 10 (1.64–58.5) | |
| Previous treatment | | | | | |
| Presentation | 27 | 20 (74) | >0.1 | 6.8 (1.64–49.7) | >0.1 |
| Recurrence | 20 | 17 (85) | | 15.4 (2–58.5) | |
| *In vitro* sensitivity | | | | | |
| Sensitive | 12 | 8 (67) | >0.1 | 2.43 (1.64–18.37) | <0.01 |
| Resistant | 35 | 29 (83) | | 13.49 (2.75–58.5) | |
| Drug | | | | | |
| Cisplatin | 24 | 20 (83) | >0.1 | 10.37 (2.64–58.5) | >0.1 |
| Carboplatin | 23 | 17 (74) | | 6.23 (1.64–51.7) | |

This increase in sensitivity was independent of sample type (ascitic fluid or biopsy) or morphology of the final cell preparation. Significant modulation by aphidicolin was seen in cells from 17 of the 19 patients with *in vitro* resistance to the platinums.

Although there was a slight increase in the incidence of modulation in the group of patients who had received previous cytotoxic therapy (85%, compared with 74% in untreated patients), this difference did not reach significance ($P>0.1$, Table I). Similarly, when looking at the frequency of modulations independent of their size, 83% of the group of patients showing *in vitro* resistance to the platinums showed significant modulation compared with 67% in the *in vitro* sensitive group ($P>0.1$).

*Sensitivity ratios*

Sensitivity ratios comparing the $LC_{50}$ for drug with and without aphidicolin were used to measure the size of the modulation effect. There was a median 10-fold (range 1.64–58.5) increase in sensitivity overall.

When patients were grouped according to *in vitro* sensitivity to the platinum drugs, there was a significant increase ($P<0.01$) in the sensitivity ratio in the resistant group (13.5-fold compared with 2.4-fold for the sensitive group, Figure 2). This was confirmed by a positive correlation between the $LC_{50}$ and the sensitivity ratio for both cisplatin ($r_s=0.505$, $n=20$, $P<0.05$) and carboplatin ($r_s=0.637$, $n=17$, $P<0.02$).

There was no significant difference in the modulating effect of aphidicolin on either cisplatin or carboplatin cytotoxicity.

### Discussion

Inhibition of DNA repair occurring after damage by platinum agents has been postulated as a method of overcoming drug resistance in ovarian cancer. Most previous studies have been conducted using established cell lines or animal tumour models. To our knowledge, this is the first report of the *in vitro* effect of combining aphidicolin with the platinum agents in fresh cells from human ovarian tumours.

**Figure 2** The range of sensitivity ratios ($LC_{50}$ of drug alone/$LC_{50}$ for drug+aphidicolin) obtained. There was a significant increase in the median of the group of patients found resistant *in vitro* to platinum compared with those found sensitive (from a 2.4-fold to a 13.5-fold increase in sensitivity), $P<0.01$.

We found that cells harvested from 22 of 25 patients showed markedly increased cytotoxicity to the platinum drugs on co-incubation with aphidicolin. It has been widely reported that dose escalation of platinum treatment leads to improved response rates in ovarian cancer (Kaye *et al.*, 1992). The modulatory approach demonstrated in this study could translate into increased response rates without elevating the dose of cytotoxic drug.

Of particular interest was the finding that the modulatory effect of aphidicolin was significantly greater in cells demonstrating *in vitro* resistance to the platinum agents, suggesting the presence of enhanced DNA repair capability. Contrary to the report from another group using established ovarian cancer cell lines (Eastman and Schulte, 1988), we found a positive correlation between the level of *in vitro* platinum resistance and the fold increase in sensitivity on co-incubation with aphidicolin. These results suggest that, as cells become more resistant there is an enhanced capacity to repair DNA lesions, allowing an increased modulatory effect through the inhibition of DNA polymerase activity.

There is clear evidence in the literature of differing degrees of platinum resistance modulation by aphidicolin both *in vitro* (Masuda *et al.*, 1988; Dempke *et al.*, 1991) and in *in vivo* murine models (Damia *et al.*, 1992; O'Dwyer *et al.*, 1994). So it appears that results vary according to cell line and, indeed, it has already been postulated that these variations may be related to DNA repair potential (Perez *et al.*, 1993). Our results using fresh cells from individual patients further confirm this hypothesis.

Not all the primary tumours tested showed significantly improved platinum cytotoxicity on co-incubation with aphidicolin, with 21% of cases showing no modulatory effect. Perhaps these cells do not have an enhanced DNA repair capacity and other mechanisms of drug resistance are involved. *In vitro* screening before treatment could help identify the group of patients who may benefit from the combination regimen. It was interesting to note that aphidicolin was equally effective in modulating resistance to cisplatin and carboplatin, thus confirming the sensitivity of these *in vitro* chemosensitivity assays.

A note of caution should always be applied when extrapolating *in vitro* experiments to the clinical situation. The final cell preparations contained a heterogeneous mixture of tumour cells with attendant stromal cells produced in response to the tumour. However, it has been repeatedly shown that DNA adduct formation in response to platinum therapy is similar in normal cells and tumour cells (Reed *et al.*, 1987; Hengstler *et al.*, 1992). Indeed, the measurement of adducts in WBCs has been suggested as an *in vitro* measure of clinical response to therapy. Another potential shortcoming is the use of largely non-dividing cells for the study of DNA damage and repair, most of the cells from these samples being quiescent, recruitable cells. However, DNA adducts are formed in both dividing and non-dividing cells on exposure to platinum (Eastman, 1990). Furthermore, as only a small proportion of cells from primary tumours are actively cycling *in vivo* (Parkinson, 1996), these *in vitro* experiments may be more clinically relevant than those using actively dividing cell lines.

Another reason why we might be finding increased positive effects compared with some previous studies is that we were testing the compound itself, not the glycinate ester. It has been reported, however, that their effects are similar *in vitro* (Damia *et al.*, 1992).

The concentration of aphidicolin required for *in vitro* modulation is clinically achievable (Sessa *et al.*, 1991), suggesting that this effect may indeed translate to the clinical situation. However, toxicity may also be increased to unacceptable levels by this approach. Given that the major toxic effect of carboplatin is myelosuppression, it may be possible to alleviate this with peripheral blood stem cell transplantation; a procedure that is now routinely carried out by many units. Furthermore, our results show increased modulation in cells from patients with *in vitro* resistance to platinum. Perhaps, enhanced DNA repair may be a feature of 'resistant' tumour cells rather than 'resistant' normal peripheral blood mononuclear cells from the same individual.

Further studies to measure DNA adduct formation in these cells from primary tumours along with normal WBCs from the same individuals after incubation in platinum agents with and without aphidicolin could prove interesting. Also, the measurement of the rate of removal of these adducts may help in the interpretation of these results. However, a recent study looking at the potentiation of temozolomide by poly(ADP-ribose) polymerase inhibitors found a disparity between the effect of the inhibitors on cell survival and their effect on DNA strand break repair. A higher concentration of inhibitor was required to affect strand break levels after exposure to temozolomide than was required to increase cytotoxicity. The authors concluded that this polymerase may be involved in DNA damage-inducible responses and so the concentration required to inhibit these differing actions may vary (Boulton *et al*., 1995). This suggests that the measurement of effect on cytotoxicity is the most specific option.

Modulation of other mechanisms of cellular resistance to the platinum drugs in primary tumours, e.g. through the glutathione pathway or mediation of oncogene expression, has shown only limited success both *in vitro* (Sargent *et al*., 1994*b*) and when applied to the clinic (Morgan *et al*., 1995; O'Dwyer *et al*., 1996). If a resistance modulation approach is going to make a significant contribution to the treatment of this devastating disease, alternative and more effective modulating agents such as aphidicolin must make the transition to the clinic.

**Acknowledgements**
This study was funded by the Haematology Research Fund, Pembury Hospital, and the BUPA Medical Foundation.

### References

BEKETIC-ORESKOVIC L AND OSMAK M. (1995). Modulation of resistance to cisplatin by amphotericin B and aphidicolin in human larynx carcinoma cells. *Cancer Chemother. Pharmacol.*, **35**, 327–333.

BOULTON S, PEMBERTON LC, PORTEOUS JK, CURTIN NJ, GRIFFIN RJ, GOLDING BT AND DURKACZ BW. (1995). Potentiation of temozolomide-induced cytotoxicity: a comparative study of the biological effects of poly(ADP-ribose) polymerase inhibitors. *Br. J. Cancer*, **72**, 849–856.

CHAO CC-K. (1994). Enhanced excision repair of DNA damage due to *cis*-diamminedichloroplatinum(II) in resistant cervix carcinoma HeLa cells. *Eur. J. Pharmacol.*, **268**, 347–355.

DAMIA G, TAGLIABUE G, ZUCCHETTI M, DAVOLI E, SESSA C, CAVALLI F AND D'INCALCI M. (1992). Activity of aphidicolin glycinate alone and in combination with cisplatin in a murine ovarian tumour resistant to cisplatin. *Cancer Chemother. Pharmacol.*, **30**, 459–464.

DEMPKE WCM, SHELLARD SA, FICHTINGER-SCHEPMAN AMJ AND HILL BT. (1991). Lack of significant modulation of the formation and removal of platinum-DNA adducts by aphidicolin glycinate in two logarithmically-growing ovarian tumour cell lines *in vitro*. *Carcinogenesis*, **12**, 525–528.

EASTMAN A. (1990). Activation of programmed cell death by anticancer agents: cisplatin as a model system. *Cancer Cells*, **2**, 275–280.

EASTMAN A AND SCHULTE N. (1988). Enhanced DNA repair as a mechanism of resistance to *cis*-diamminedichloroplatinum(II). *Biochemistry*, **27**, 4730–4734.

FRIEDLANDER ML. (1992). Drug resistance in ovarian cancer. *Int. J. Gynecol. Cancer*, (suppl. 1), 2–9.

GORMLEY PE, BULL JM, LEROY AF AND CYSYK R. (1979). Kinetics of *cis*-dichlorodiammineplatinum. *Clin. Pharmacol. Ther.*, **23**, 351–357.

HENGSTLER JG, FUCHS J AND OESCH F. (1992). DNA strand breaks and DNA cross-links in peripheral mononuclear cells of ovarian cancer patients during chemotherapy with cyclophosphamide/carboplatin. *Cancer Res.*, **52**, 5622–5626.

HILLS CA, KELLAND LR, ABEL G, SIRACKY J, WILSON AP AND HARRAP KR. (1989). Biological properties of ten human ovarian carcinoma cell lines: calibration *in vitro* against four platinum complexes. *Br. J. Cancer*, **59**, 527–534.

KAYE SB. (1993). Chemotherapy for ovarian cancer. *Eur. J. Cancer*, **29**, 632–635.

KAYE SB, LEWIS CR, PAUL J, DUNCAN D, GORDON HK, KITCHENER HC, CRUICKSHANK DJ, ATKINSON RJ, SOUKOP M, RANKIN EM, CASSIDY J, DAVIS JA, REED NS, CRAWFORD SM, MacLEAN A, SWAPP GA, SARKER TK, KENNEDY JH AND SYMONDS RP. (1992). Randomised study of two doses of cisplatin with cyclophosphamide in epithelial ovarian cancer. *Lancet*, **340**, 329–333.

MASUDA H, OZOLS RF, LAI G-M, FOJO A, ROTHENBERG M AND HAMILTON T. (1988). Increased DNA repair as a mechanism of acquired resistance to *cis*-diamminedichloroplatinum(II) in human ovarian cancer cell lines. *Cancer Res.*, **48**, 5713–5716.

MORGAN RJ, MARGOLIN K, RASCHKO J, AKMAN S, LEONG L, SOMLO G, SCANLON K, AHN C, CARROLL M AND DOROSHOW JH. (1995). Phase I trial of carboplatin and infusional cyclosporine in advanced malignancy. *J. Clin. Oncol.*, **13**, 2238–2246.

NEWELL DR, SIDDICK ZH, GUMBRELL LA, BOXALL FE, GORE ME, SMITH IE AND CALVERT AH. (1987). Plasma free platinum pharmacokinetics in patients treated with high dose carboplatin. *Eur. J. Cancer Clin. Oncol.*, **23**, 1399–1405.

O'DWYER PJ, MOYER JD, SUFFNESS M, HARRISON SD, CYSYK R, HAMILTON TC AND PLOWMAN J. (1994). Antitumor activity and biochemical effects of aphidicolin glycinate (NSC 303812) alone and in combination with cisplatin *in vivo*. *Cancer Res.*, **54**, 724–729.

O'DWYER PJ, HAMILTON TC, LACRETA FP, GALLO JM, KILPATRICK D, HALBHERR T, BRENNAN J, BOOKMAN M, HOFFMAN J, YOUNG RC, COMIS RL AND OZOLS RF. (1996). Phase I trial of buthionine sulfoximine in combination with melphalan in patients with cancer. *J. Clin. Oncol.*, **14**, 249–256.

OZOLS RF. (1992). Chemotherapy for advanced epithelial ovarian cancer. *Haematol. Oncol. Clin. N. Am.*, **6**, 879–894.

PARKINSON EK. (1996). Do telomerase antagonists represent a novel anti-cancer strategy? *Br. J. Cancer*, **73**, 1–4.

PEREZ RP, HAMILTON TC, OZOLS RF AND YOUNG RC. (1993). Mechanisms and modulation of resistance to chemotherapy in ovarian cancer. *Cancer* (suppl.), **71**, 1571–1580.

REED E, OZOLS R, TARONE R, YUSPA SH AND POIRIER MC. (1987). Platinum DNA adducts in leukocyte DNA correlate with disease response in ovarian cancer patients receiving platinum-based chemotherapy. *Proc. Natl Acad. Sci. USA*, **84**, 5024–5028.

SARGENT J, ELGIE A, TAYLOR CG, WILSON J, ALTON P AND HILL JG. (1994*a*). The identification of drug resistance in ovarian cancer and breast cancer: application of the MTT assay. In *Chemosensitivity Testing in Gynecologic Malignancies and Breast Cancer. Contrib. Gynecol. Obstet*, vol 19, Kochli OR, Sevin B-U and Haller U. (eds) pp. 64–75, Karger: Basle.

SARGENT J, TAYLOR C, ELGIE A AND WILLIAMSON C. (1994*b*). *In vitro* modification of drug resistance in fresh tumor cells from patients with ovarian cancer. *Anti-Cancer Drugs*, **5** (suppl. 1), 61.

SESSA C, ZUCCHETTI M, DAVOLI E, CALIFANO R, CAVALLI F, FRUSTACI S, GUMBRELL L, SULKES A, WINOGRAD B AND D'INCALCI M. (1991). Phase I and clinical evaluation of aphidicolin glycinate. *J. Natl Cancer Inst.*, **83**, 1160–1164.

WILSON JK, SARGENT JM, ELGIE AW, HILL JG AND TAYLOR CG. (1990). A feasibility study of the MTT assay for chemosensitivity testing in ovarian malignancy. *Br. J. Cancer*, **62**, 189–194.